# Characterization of the immune cell landscape in CRC: Clinical implications of tumour-infiltrating leukocytes in early- and late-stage CRC

**DOI:** 10.3389/fimmu.2022.978862

**Published:** 2023-02-08

**Authors:** Zainab Ali Bazzi, Sophie Sneddon, Peter G. Y. Zhang, Isabella T. Tai

**Affiliations:** ^1^Division of Gastroenterology, Department of Medicine, University of British Columbia, Vancouver, BC, Canada; ^2^Canada’s Michael Smith Genome Sciences Centre, BC Cancer, Vancouver, BC, Canada

**Keywords:** colorectal cancer, prognosis, dendritic cells, T cells, cancer immunology

## Abstract

**Introduction:**

Colorectal cancer (CRC) is the third leading cause of cancer-related deaths globally. Tumour-infiltrating leukocytes play an important role in cancers, including CRC. We therefore sought to characterize the impact of tumour-infiltrating leukocytes on CRC prognosis.

**Methods:**

To determine whether the immune cell profile within CRC tissue could influence prognosis, we employed three computational methodologies (CIBERSORT, xCell and MCPcounter) to predict abundance of immune cell types, based on gene expression. This was done using two patient cohorts, TCGA and BC Cancer Personalized OncoGenomics (POG).

**Results:**

We observed significant differences in immune cell composition between CRC and normal adjacent colon tissue, as well as differences in based on method of analysis. Evaluation of survival based on immune cell types revealed dendritic cells as a positive prognostic marker, consistently across methodologies. Mast cells were also found to be a positive prognostic marker, but in a stage-dependent manner. Unsupervised cluster analysis demonstrated that significant differences in immune cell composition has a more pronounced effect on prognosis in early-stage CRC, compared to late-stage CRC. This analysis revealed a distinct group of individuals with early-stage CRC which have an immune infiltration signature that indicates better survival probability.

**Conclusions:**

Taken together, characterization of the immune landscape in CRC has provided a powerful tool to assess prognosis. We anticipate that further characterization of the immune landscape will facilitate use of immunotherapies in CRC.

## Background

Colorectal cancer (CRC) is the third leading cause of cancer-related deaths worldwide (1). While screening strategies and therapeutics have improved outcomes for colorectal cancer over the past few decades, prognosis for advanced stages remains very poor (2). CRC is conventionally classified by clinicopathological characteristics, such as TNM stage and histology (3, 4). Unfortunately, these features are often ambiguous in predicting clinical outcomes and response to therapeutics. In an attempt to circumvent this, CRC is often further characterized by genomic factors such as microsatellite instability status, as well as BRAF and KRAS mutational status (4, 5). While these factors are collectively better able to predict prognosis and drug response, they are limited and heterogeneity in clinical outcome remains problematic. Therefore, there is an urgent need for further identification of genomic and phenotypic features for the development of more effective prognostics and therapeutics for CRC.

Recent studies have sought to understand the complex relationship between the immune system and cancer. Importantly, studies have demonstrated that tumour progression and prognosis are influenced by immune cell infiltration in tumours and their surrounding tissues. Specifically, lymphocytic infiltration has been associated with good prognosis in breast, lung, prostate, ovarian and CRC (6–9). Due to this correlation, it has become evident that exploiting the immune system is not only a viable therapeutic strategy, but also may prove to be effective for prognostic purposes.

Several studies have identified tumour-infiltrating lymphocytes in CRC (9–11). These studies have shown that lymphocytic infiltration of CRC is associated with more favourable prognosis. Furthermore, a study by Galon et al. demonstrated that immunological infiltration of CRC is a better prognostic indicator than conventional histopathological staining (12). Additionally, infiltration of CRC with memory T cells was shown to be inversely correlated with signs of early metastatic invasion (13, 14). Collectively, these factors identify CRC as a promising target for immunotherapy. However, while immunotherapy is approved for a number of cancer types (14–16), clinical trials in unselected patients with CRC have not yielded promising clinical outcomes (17, 18). Currently, approval of checkpoint inhibition immunotherapy for CRC is limited to a subset of patients with microsatellite instability-high (MSI-H) or mismatch repair deficient (dMMR) tumours (19). While MSI-H or dMMR CRC, response to checkpoint inhibitor immunotherapy is encouraging, these subtypes only account for approximately 15% of CRC cases (20). It is therefore evident that further studies are necessary to identify additional factors that influence response to immunotherapy treatment in CRC.

Traditional methods used to identify infiltrated immune cells include immunohistochemistry and flow cytometry. Unfortunately, both of these methods are limited in their ability to accurately identify subsets of immune cells within a bulk tumour (14, 21). Several novel methods have been employed using gene expression profiles to predict abundance of specific immune cells within a bulk tumour. Microenvironment Cell Populations-counter (MCP-counter), is a method use to quantify the absolute abundance of eight immune and two stromal cell types (22). MCP-counter uses gene expression data to generate an abundance score for B lymphocytes, cytotoxic lymphocytes, CD3+ T cells, CD8+ T cells, monocytic lineage cells, myeloid dendritic cells, neutrophils, NK cells, endothelial cells and fibroblasts (22).

CIBERSORT is a deconvolution method used to identify subsets of infiltrated immune cells within a tumour and determine correlations to clinical outcomes (14, 21, 23). CIBERSORT uses relative gene expression of 547 genes that distinguishes 22 human hematopoietic cell types (referred to as a leukocyte gene signature matrix, LM22). LM22 is specifically able to phenotypically distinguish B cells, dendritic cells, eosinophils, macrophages, mast cells, monocytes, neutrophils, natural killer cells, plasma cells and T cells. xCell is a gene-signature based method, which uses a combination of gene set enrichment with deconvolution to analyze microarray and RNA-seq expression profiles (24). This method is able to predict abundance of 64 cell types, including immune cells, hematopoietic cells, and epithelial cells. Specifically, xCell can also generate abundance scores for adaptive and innate immune cells, including T cells, B cells, macrophages, monocytes, neutrophils, dendritic cells and natural killer cells (24). In this study, we employ MCP-counter, CIBERSORT and xCell to assess the immune cell composition in CRC and identify an immune profile associated with improved outcomes in survival.

## Materials and methods

### Data Mining

For the exploration datasets, publicly available expression data were downloaded from The Cancer Genome Atlas (TCGA) up to June 31, 2018 with the Genomic Data Commons (GDC) application. The data, which consist of RNA-sequencing data of 644 tumours and 51 adjacent normal tissues from CRC patients, were generated using the Illumina HiSeq platform. Clinical data from these patients were also retrieved from TCGA. To compare primary tumours with metastatic tumours, expression data and clinical data were retrieved from BC Cancer Personalized OncoGenomics (POG) Program on September 10, 2018, which consisted of RNA-sequencing data of 73 tumours from metastatic CRC patients.

### Determination of tumour-infiltrating immune profile

The R-script for the CIBERSORT algorithm was downloaded from https://cibersort.stanford.edu/ and run under R version 3.4.1 environment (21, 23). Immune profiles of 22 types of infiltrating immune cells were determined with CIBERSORT using the default signature matrix (designated as “LM22” by the authors) at 1000 permutations. Quantile normalization was turned off for the exploration TCGA dataset consisting of RNA-sequencing data (21). The default signature matrix containing 547 immune marker genes was used to characterize immune cell composition of 22 immune cell types. The gene annotation was examined to ensure nomenclature consistency between TCGA and CIBERSORT. Eleven genes were renamed with the most recent gene ID published on HUGO Gene Nomenclature Committee (HGNC, https://www.genenames.org/); five genes or ncRNAs (*GSTT1*, *LILRA3*, *LINC00597*, *LOC100130100*, *LOC126987*) were removed from the matrix due to their retirement in the latest human genome annotation (hg38) which was used for the TCGA expression data. As a summary, CIBERSORT provides a *P*-value for each sample using Monte Carlo sampling, indicating its significance level in the results (21).

Deconvolution to identify immune cell subsets was also performed using xCell and MCPcounter (22, 24). xCell uses an ssGSEA approach to quantify the enrichment of gene signatures for 64 immune and stromal cell subsets, while MCPcounter quantifies the abundance of 8 immune and 2 non-immune stromal populations in heterogeneous tissue samples. All downstream analyses were performed using CIBERSORT, xCell and MCPcounter deconvolution results.

### Comparative Analyses of TCGA data

RNA-sequencing data from a total of 644 tumour samples and 51 adjacent normal samples were run through the CIBERSORT algorithm. A subset of 308 out of 644 (~48%) tumour samples and 40 out of 51 (~78%) adjacent normal samples, passed CIBERSORT analysis with *P*-value <0.05 and were used for downtown analyses. Comparative analyses for relative percentage of immune cells, total number of infiltrating immune cells as well as individual gene expression level were done with Wilcoxon signed-rank test (between two groups) for normal *vs.* CRC groups. Five levels of significance were used in comparative analyses (and subsequently used in figures), determined by *P*-values: “ns” (not significant) “*” for p < 0.05, “**” for p < 0.01, “***” for p < 0.001 and “****” for p < 0.0001.

### Survival analyses of TCGA data

Survival curves were generated and plotted with R package “Survminer” (version 0.4.3) (25). For comparative survival analyses, the threshold for high and low cell fractions or expression levels were determined with ROC curve, as previously described (26), and the log-rank test was applied to analyze differences among groups. Statistics such as hazard ratio, mean survival and confident intervals were extracted from the survival objects constructed with R package “survival” (version 2.42-6) (27).

### Analyses of POG data

The POG dataset (metastatic cohort), which consisted of expression and clinical data from 73 metastatic CRC samples, were re-formatted to match the format of the TCGA expression and clinical data, which was then used as input for the bioinformatic pipeline that was used for the TCGA exploration dataset. The same filtering step for CIBERSORT, xCell and MCP analyses were used and 58 samples that passed the p<0.05 in CIBERSORT analysis were used for downstream expression, survival and other comparative analyses, as described previously for the TCGA dataset. As no p-value is provided with xCell and MCPcounter, all results were used for downstream analyses using these deconvolution methods.

### Clustering of CRC samples based on immune profile

To identify subtypes of CRC tumours based on immune profiles, unsupervised k-means clustering analysis was completed utilizing all CRC tumour samples. This heuristic algorithm uses the centroid principle, which is used on a geometric centre of a cluster and will minimize the distance between a point and a centroid to assign this point to a cluster (28). First, we computed the optimal number of clusters (the k value) that would be best attributed to the TCGA data; a silhouette analysis was carried out to determine the inter-cluster distances, which informs the relative distances of each cluster to the others. A simulation silhouette analysis for several k values (k = 1, k = 2… k = 10) was conducted (29), and the most significant value corresponded to k = 2 for an average silhouette score of 0.09. Once all CRC tumour samples (N = 308) were attributed to two clusters, the relative number of immune cells were compared between the two clusters (Cluster 1 with 142 samples; Cluster 2 with 166 samples). Supervised clustering was then performed using immune cells that significantly differed between the two clusters that emerged from the unsupervised clustering. These cell types were used as attributors, which refined the two clusters with minimal number of tumour samples in the overlapping region between the two clusters (Cluster 1 with 141 samples; Cluster 2 with 167 samples).

Differential gene expression analysis was performed on the clusters identified in the TCGA and POG data using DESeq2 in R (v.1.28.1) (30). Functional annotation of the resulting gene sets was performed using clusterProfiler in R (v.3.16.1) (31). Significance was determined using an adjusted p-value cutoff of 0.1 for both methods.

### Univariate and multivariate survival analyses and statistics

To identify genes important for prognosis of CRC patients based on immune profiles, univariate and multivariate survival analyses were performed using significantly differentially expressed genes identified in the previous section. In addition to the log-rank test described previously, the univariate random forests analysis was also used to determine if a gene could play a role in the survival of CRC patients. The R package, ranger, implemented a high performance random forest method based on survival analyses (version 0.10.) (32). Statistical significances for the output (the variable: importance score) was measured based on the empirical null distribution as described previously (33). For the multivariate counterpart of survival analysis, the multivariate Cox-model was used to compare relative contributions of different factors, such as immune cell types or candidate genes. Hazard ratio, mean survival time and confidence interval values based on the log-rank test and Cox-model were extracted from the survival objects constructed in the R environment during univariate and multivariate survival analyses with the packages described above.

### Comparison of immune profiling between early- and late-stage CRC

The clinical data on tumour stage was obtained from the TCGA CRC clinical database. The early-stage CRC cohort contained 169 (~55% of the total 308 samples) samples from stage I and II tumours; and the late-stage CRC cohort contained 127 (~41%) samples from stage III and IV tumours. The remaining 12 samples (~4%) did not have their tumour stage specified and were excluded from the early- vs. late-stage CRC analyses. Immune profiles, cluster, expression and survival analyses were subsequently repeated for the early- and late-stage CRC cohorts, following the same methods that were used for the entire CRC dataset, as described previously. The results for each analysis were compared among the entire CRC dataset, the early-stage CRC and the late-stage CRC cohorts.

## Results

### Immune cell composition in CRC

**Figure 1:**
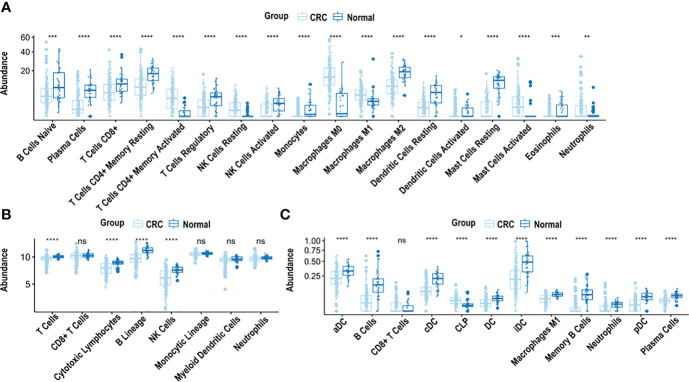
*Analysis of immune cell composition of CRC samples using CIBERSORT, xCell and MCP-counter.* **(A)** Immune cell abundance in CRC tissue compared to normal adjacent colon tissue, based on CIBERSORT analysis. **(B)** Immune cell abundance in CRC tissue compared to normal adjacent colon tissue, based on MCP-counter analysis. **(C)** Immune cell abundance in CRC tissue compared to normal adjacent colon tissue, based on xCell analysis. CRC was compared to normal tissue using the Wilcoxon test. ****p < 0.0001, ***p < 0.001, **p < 0.01, *p < 0.05, n.s. non-significant.

CIBERSORT, xCell and MCP-counter revealed that the immune cell landscape in CRC is distinctly different from normal tissues (**Figure 1**).

CIBERSORT, used to estimate cell fractions of 22 immune cell types in CRC and normal adjacent colon tissue, demonstrated differences in relative immune cell composition in CRC, compared to adjacent normal tissue, (**Figure 1A**). Cell abundance is a quantitative measure of the cell composition, based on the gene expression. Specifically, there was an abundance of M2 macrophages, resting dendritic cells, resting mast cells, monocytes, eosinophils, activated natural killer cells, CD8^+^ T cells, regulatory T cells, CD4^+^ memory resting T cells and plasma cells in adjacent normal colon tissue, compared to CRC (p < 0.0001), (**Figure 1A**). In contrast, an abundance of M0 macrophages, M1 macrophages, resting natural killer cells, activated mast cells, and CD4^+^ memory activated T cells was observed in CRC (p < 0.0001), compared to adjacent normal colon tissue (**Figure 1A**).

MCP-counter was used to quantify abundance of 8 immune cell types in CRC and adjacent normal colon tissue. B cells, cytotoxic lymphocytes, natural killer cells and T cells were significantly more abundant in adjacent normal colon compared to CRC (p < 0.0001) (**Figure 1B**). xCell examined the abundance of 64 cell types, of which 35 were characterized as immune cells. Significant differences were observed in the immune cell composition in CRC *vs*. adjacent normal colon tissue. Specifically, abundance of B cells, memory B cells, plasma cells, neutrophils and M1 macrophages were significantly higher in adjacent normal colon tissue *vs.* CRC (p < 0.0001) (**Figure 1C**). Additionally, abundance of several types of dendritic cells including activated, conventional, plasmacytoid, and immature dendritic cells were significantly higher in adjacent normal colon *vs.* CRC (p < 0.0001) (**Figure 1C**). Furthermore, common lymphoid progenitor cells (CLP) were significantly higher in CRC *vs.* adjacent normal colon tissue (p < 0.0001) (**Figure 1C**).

**Figure 2:**
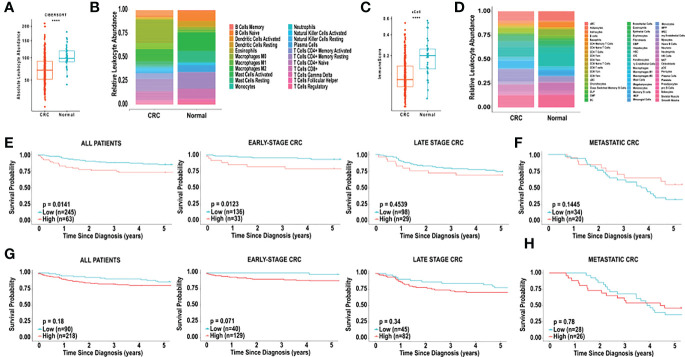
*Analysis of immune cell composition and survival of CRC using CIBERSORT and xCell.* **(A)** Relative leukocyte fractions of 22 cell types were determined using CIBERSORT for 308 CRC tumours and 40 normal adjacent colon tissue samples. Absolute leukocyte abundance was determined by the sum of all immune cell infiltration, based on CIBERSORT, for CRC tissue and normal adjacent colon tissue. Each dot represents one patient. **(B)** Relative leukocyte fractions are depicted for CRC tissue and normal adjacent colon tissue, as predicted by CIBERSORT. **(C)** The Immunoscore, as determined by xCell, for CRC tissue and normal adjacent colon tissue. **(D)** Relative leukocyte fractions are depicted for CRC tissue and normal adjacent colon tissue, as predicted by xCell. **(E)** Kaplan-Meier curves for overall five-year survival based on the CIBERSORT absolute leukocyte abundance for all TCGA patients, patients with early-stage CRC, late-stage CRC and **(F)** metastatic CRC. **(G)** Kaplan-Meier curves for overall five-year survival based on the xCell Immunoscore for all TCGA patients, patients with early-stage CRC, late-stage CRC and **(H)** metastatic CRC. CRC was compared to normal tissue using the Wilcoxon test. ****p < 0.0001. Groups with high and low cell numbers were compared with log-rank test.

Unlike MCP-counter, CIBERSORT and xCell both generate total immune cell infiltration scores, based on the abundance of immune cells within each sample. Specifically, the absolute leukocyte abundance from the CIBERSORT analysis, and the Immunoscore from xCell are both values representing the total immune cell infiltration within a patient sample. As shown in **Figure 2A**, the absolute leukocyte abundance for normal colon was significantly higher compared to CRC (p < 0.0001). Similarly, the Immunoscore for normal colon was significantly higher compared to CRC (p < 0.0001), as shown in **Figure 2C**.

### Overall survival based on total immune cell infiltration of individuals with CRC

Based on the CIBERSORT analysis, we found that the absolute leukocyte abundance was significantly correlated with lower 5-year survival (HR = 2.0224, p = 0.01408), as shown in **Figure 2E**. Furthermore, we examined the effect of total immune cell infiltration on tumour stage at diagnosis. The 5-year survival of patients with early-stage CRC (TCGA, stages I and II) was inversely correlated with lower immune cell infiltration (HR = 3.2091, p = 0.01228), and this effect was no longer observed with late-stage CRC (TCGA, stages III and IV) (HR = 1.3363, p = 0.4539) (**Figure 2E**) or metastatic (POG cohort) (HR = 0.5691, p = 0.1445) (**Figure 2E**).

We next examined the correlation between the “Immunoscore” observed from the xCell analysis, and found that the survival did not correlated with overall 5-year survival in all patients with CRC (HR = 1.5425, p = 0.1820, **Figure 2F**). Examination of the effect of the Immunoscore on overall 5-year survival did not reveal any significant correlations based on tumour stage. Specifically, in early-stage CRC, high Immunoscore was non-significantly associated with poor overall 5-year survival (HR = 5.2825, p = 0.0705, **Figure 2F**). In late-stage and metastatic CRC no significant associations in overall 5-year survival were observed, based on Immunoscore (**Figure 2F**).

### Overall survival based on immune cell infiltration of individuals with CRC

**Figure 3:**
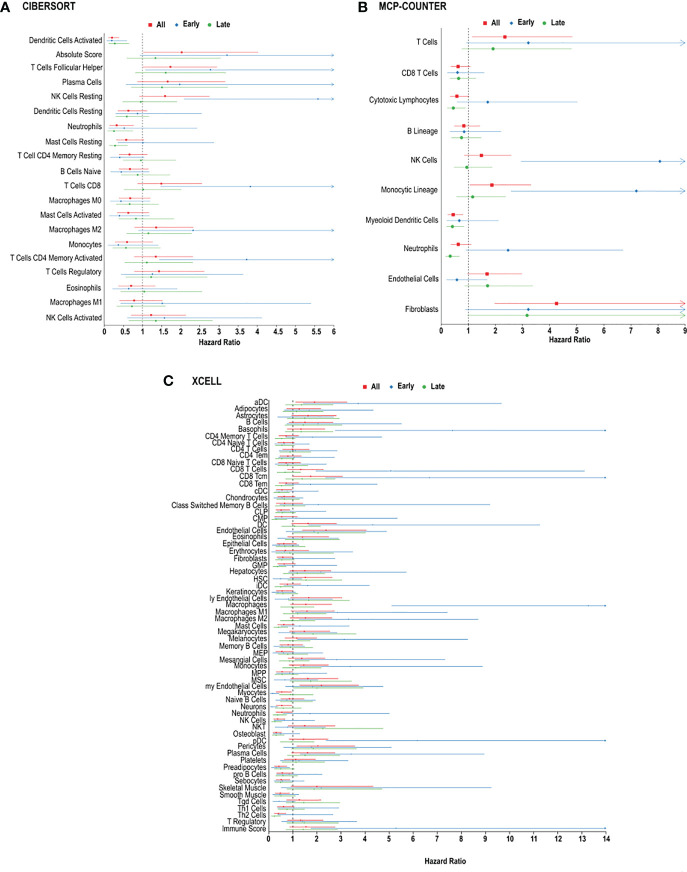
*Immune cell infiltration and survival analysis of CRC.* **(A)** Analysis of prognostic impact of immune cell infiltration in all patients, patients with early-stage CRC and late-stage CRC, based on the CIBERSORT analysis. **(B)** Analysis of prognostic impact of immune cell infiltration in all patients, patients with early-stage CRC and late-stage CRC, based on the MCP-counter analysis. **(C)** Analysis of prognostic impact of immune cell infiltration in all patients, patients with early-stage CRC and late-stage CRC, based on the xCell analysis.

We next performed random forest modeling to show an association between overall survival and immune cell infiltration (**Figure 3**). We identified two cell types, in the CIBERSORT analysis, that were significantly associated with overall survival (**Figure 3A**).

In CRC, regardless of stage, CIBERSORT, activated dendritic cells (HR = 0.2013 [0.1064-0.3809], p = 0.002714) and follicular helper T cells (HR = 1.7281 [1.0136-2.9460], p = 0.04923) were associated with overall survival in all CRC patients (**Figure 3A**). MCP-counter found cytotoxic lymphocytes (HR = 0.5825 [0.3285-1.0329], p = 0.0456), myeloid dendritic cells (HR = 0.4457 [0.2457-0.8085], p = 0.0023) and T cells (HR = 2.3514 [1.1431-4.8367], p = 0.0026) were associated with improved 5-year survival, while xCell analysis linked activated dendritic cells (HR = 1.9021 [1.1144-3.2467], p = 0.0255), common lymphoid progenitors (CLP) (HR = 0.5132 [0.3010-0.8749], p = 0.0185), neutrophils (HR = 0.5437 [0.3016-0.9802], p = 0.0244), natural killer (NK) cells (HR = 0.3684 [0.1995-0.6801], p = 0.0162), and type-2 helper T (Th2) cells (HR = 0.4034 [0.2289-0.7109], p = 0.0100), with better prognosis (**Figure 3C**).

In order to determine if specific immune cell types differ in early *vs.* late stage CRC, we used the 3 algorithms to assess the immune landscape in early (stage I + II) *vs.* late (stage III + IV). Large fractions of resting natural killer cells (HR = 5.5870, p = 0.00068) and memory activated CD4^+^ T cells (HR = 3.7234, p = 0.01352) were associated with poor overall survival in patients, using CIBERSORT analysis (**Figure 3B**). Similarly, using MCP-counter, patients with early-stage CRC, poor overall survival was found in individuals with, natural killer cells (HR = 8.0681, p = 0.01572), and T cells (HR = 3.2172, p = 0.01207) (**Figure 3B**). MCP-counter analysis also showed large fractions of monocytic lineage cells (HR = 7.2005, p = 0.02490) to be associated with poor overall survival in patients with early-stage CRC. Interestingly, xCell analysis revealed similar results: monocytes (HR = 3.3891, p = 0.04125), M1 macrophages (HR = 2.8569, p = 0.0389), M2 macrophages (HR = 3.3126, p = 0.0457) and plasma cells (HR = 3.4186, p = 0.03968). (**Figure 3C**). Similarly, were indicative of poor overall survival in individuals with early-stage CRC, based on xCell analysis (**Figure 3C**).

In late stage CRC, high resting mast cells (HR = 0.02812 [0.1302-0.6075], p = 0.02481) was associated with better overall survival, based on CIBERSORT analysis (**Figure 3C**), and as well xCellmast cells (HR = 0.4052, p = 0.01610). Using MCP-counter, patients with late-stage CRC, large fractions of cytotoxic lymphocytes (HR = 0.4500, p = 0.01849), and neutrophils (HR = 0.3349, p = 0.0011) were also associated with better overall survival (**Figure 3B**). xCell analysis also identified neutrophils (HR = 0.3614, p = 0.0021) and natural killer cells (HR = 0.2575, p = 0.01542), and Th2 cells (HR = 0.2335, p = 0.00864) were found to be significantly correlated with better overall survival (**Figure 3C**).

In patients with metastatic CRC, M2 macrophages (HR = 2.6132, p = 0.0059) and neutrophils (HR = 2.3441, p = 0.01769) were associated with poor prognosis, based on CIBERSORT analysis (**Figure 3A**). Plasma cells were found to be correlated with poor overall survival in both CIBERSORT (HR = 2.1173, p = 0.04919) and xCell analysis (HR = 2.0555, p = 0.0058). Additionally, in patients with metastatic CRC, large fractions of helper T cells (HR = 0.2013, p = 0.002714), and activated mast cells (HR = 0.2013, p = 0.002714) were associated with better overall survival (**Figure 3A**), using CIBERSORT analysis, however mast cells (HR = 2.7450, p = 0.01318) and Th2 cells (HR = 2.6543, p = 0.0058) were found to be inversely correlated with overall survival, using xCell analysis (**Figure 3C**).

### Overall survival based on dendritic cell infiltration in individuals with CRC

**Figure 4:**
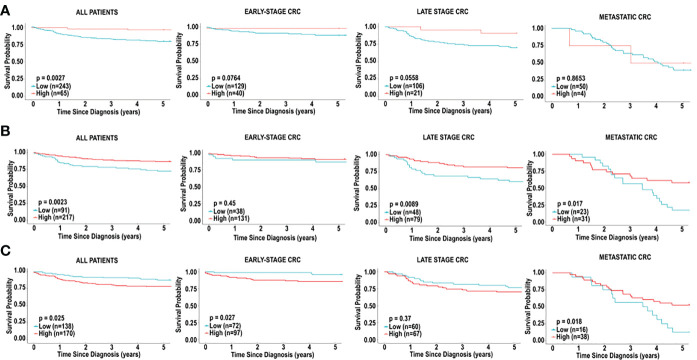
*Immune cell infiltration and survival analysis of CRC based on activated dendritic cells.* **(A)** Kaplan-Meier curve for overall five-year survival based on relative cell fractions of activated dendritic cells, based on CIBERSORT analysis for all patients, patients with early-stage CRC, late-stage CRC and metastatic CRC. **(B)** Kaplan-Meier curve for overall five-year survival based on relative cell fractions of activated dendritic cells, based on MCP-counter analysis for all patients, patients with early-stage CRC, late-stage CRC and metastatic CRC. **(C)** Kaplan-Meier curve for overall five-year survival based on relative cell fractions of activated dendritic cells, based on xCell analysis for all patients, patients with early-stage CRC, late-stage CRC and metastatic CRC. Groups with high and low cell numbers were compared with log-rank test.

**Figure 5:**
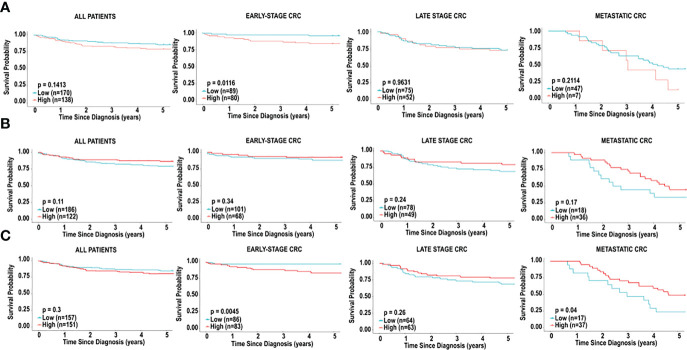
*Immune cell infiltration and survival analysis of CRC based on CD8^+^ T cells.* **(A)** Kaplan-Meier curve for overall five-year survival based on relative cell fractions of CD8^+^ T cells, based on CIBERSORT analysis for all patients, patients with early-stage CRC, late-stage CRC and metastatic CRC. **(B)** Kaplan-Meier curve for overall five-year survival based on relative cell fractions of CD8^+^ T cells, based on xCell analysis for all patients, patients with early-stage CRC, late-stage CRC and metastatic CRC, based on MCP-counter analysis. **(C)** Kaplan-Meier curve for overall five-year survival based on relative cell fractions of CD8^+^ T cells, based on xCell analysis for all patients, patients with early-stage CRC, late-stage CRC and metastatic CRC, based on MCP-counter analysis. Groups with high and low cell numbers were compared with log-rank test.

As previously mentioned, dendritic cells appeared to influence prognosis, using all 3 platforms, CIBERSORT, xCell and MCP-counter. We therefore examined the effects of low levels of dendritic cell infiltration on overall survival based in early, late stage, and metastatic CRC. Higher levels of dendritic cells in CRC were associated with improved 5-year survival in all CRC patients, based on CIBERSORT (HR = 0.2013 [0.1064-0.3809], p = 0.002714) and MCP-counter (HR = 0.4457 [0.2457-0.8085], p = 0.0023), but the opposite was observed when analyzed by xCell (HR = 1.9021 [1.1144-3.2467], p = 0.0255) (**Figure 5**). This effect was mostly due to the level of dendritic cell infiltration in late-stage CRC. We observed significant differences in the dendritic cells associated with overall survival in patients with early-stage CRC compared to patients with late-stage CRC and metastatic CRC. We also observed differences in overall survival based on the 3 prediction analyses that were used. Using CIBERSORT analysis, examination of activated dendritic cells in early-stage CRC and late-stage CRC demonstrated associations with non-significant favourable outcomes (HR = 0.1946 [0.0643-0.5895], p = 0.0764) and (HR = 0.2727 [0.1151-0.6465], p = 0.0558), respectively, as shown in **Figure 4A**. Furthermore, activated dendritic cell abundance predicted by CIBERSORT analysis in metastatic CRC did not reveal an association with overall survival (HR = 0.8836 [0.2276-3.4309], p = 0.8653), as shown in **Figure 4A**.

Furthermore, using myeloid dendritic cell abundance generated by MCP-counter, we assessed the overall survival based on tumour stage. Contrary to results obtained from CIBERSORT, no association was observed between myeloid dendritic cells and overall survival in early-stage CRC (HR = 0.6705 [0.2122-2.1189], p = 0.4495), as shown in **Figure 4B**. Additionally, myeloid dendritic cells were significantly associated with better overall survival in late-stage CRC (HR = 0.4173 [0.2058-0.8460], p = 0.0089), and metastatic CRC (HR = 0.4382 [0.2154-0.8916], p = 0.0171), as shown in **Figure 4B**.

Next, overall survival based on tumour stage was examined using activated dendritic cell abundance generated by the xCell analysis. Conflicting with results obtained from CIBERSORT and MCP-counter, high levels of activated dendritic cells were associated with poor overall survival in early-stage CRC (HR = 3.7114 [1.4254-9.6636], p = 0.02686), as shown in **Figure 4C**. Additionally, similar to results from MCP-counter, high levels of activated dendritic cells were associated with better overall survival in metastatic CRC (HR = 0.4429 [0.2203-0.9795], p = 0.0181) (**Figure 4C**). No significant impact on prognosis was observed when comparing high and low levels of activated dendritic cells in late-stage CRC, as shown in **Figure 4C**.

### Overall survival based on CD8^+^ T cell infiltration in individuals with CRC

CD8^+^ T cells have been widely demonstrated to be predictive of prognosis in cancers, including CRC (34). We decided to assess if CD8+ T cells in CRC tissue was associated with prognosis. Interestingly, in our study, CD8^+^ T cells did not emerge as a cell type significantly associated with 5-year survival in CRC patients in the TCGA cohort, irrespective of stage (**Figure 5**), in all 3 platforms (CIBERSORT, xCell and MCP-counter). However, given the significance of this cell type in literature, we further examined the significance of CD8^+^ T cells on prognosis, based on tumour stage. The 5-year survival in patients with early-stage CRC revealed better prognosis for individuals with lower fractions of CD8^+^ T cells (HR = 3.8167, 95% CI [1.4706-9.9072], p = 0.0116), based on CIBERSORT analysis (**Figure 5A**). Furthermore, we did not observe any significant differences in overall survival when comparing levels of CD8^+^ T cell in late-stage CRC (HR = 1.1063, 95% CI [0.5128-2.0140], p = 0.9631) and metastatic CRC (HR = 1.7461, 95% CI [0.5936-5.1366], p = 0.2114), as shown in **Figure 5A**.

Next, we used xCell to assess the effects of CD8^+^ T cells on overall survival, based on tumour stage. Similar to results with CIBERSORT, high levels of CD8^+^ T cells early-stage CRC were associated with significantly poor outcome in individuals with early-stage CRC (HR = 5.0638, 95% CI [1.9554-13.1134], p = 0.0045), as shown in **Figure 5B**. Additionally, as shown in **Figure 5B**, we did not observe a significant association when comparing levels of CD8^+^ T cell in late-stage CRC (HR = 0.6775, 95% CI [0.3459-1.5271], p = 0.2606). Contradictory to results with CIBERSORT, higher levels of CD8^+^ T cells were associated with better overall survival in individuals with metastatic CRC (HR = 0.4870, 95% CI [0.2188-1.0840], p = 0.0401), **Figure 5B**. Furthermore, analysis with MCP-counter did not reveal any significant associations with CD8^+^ T cells in early-stage CRC, late-stage CRC or metastatic CRC and prognosis (**Figure 5C**).

### Unsupervised clustering of patients based on immune cell infiltration

An unsupervised clustering algorithm was used to cluster the 308 samples based on immune cell composition generated by CIBERSORT, xCell and MCP-counter. Additionally, unsupervised clustering was used to cluster early-stage CRC, late-stage CRC and metastatic CRC. For each analysis, two distinct groups emerged from the clustering algorithm and 5-yearsurvival was examined for each cluster.

**Figure 6:**
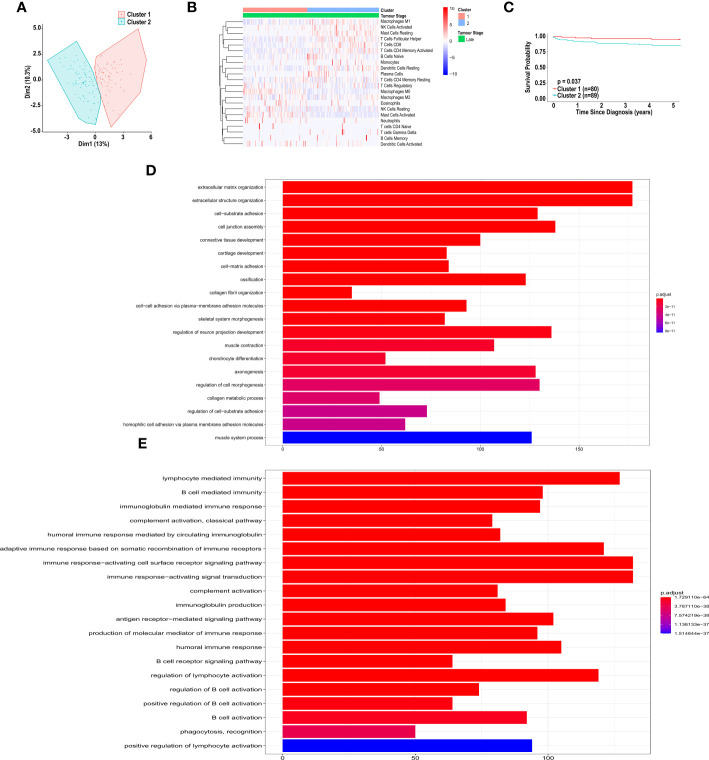
*Unsupervised clustering of patients with CRC, based on immune cell infiltration, using CIBERSORT analysis.* **(A)** Unsupervised clustering of early-stage CRC patients, based on immune cell infiltration, using CIBERSORT analysis. **(B)** Heatmap of immune cell infiltration, of patients in cluster 1 and 2, based on CIBERSORT analysis. **(C)** Kaplan-Meier curve for overall five-year survival for clusters 1 and 2, resulting from unsupervised clustering for early-stage CRC patients, based on CIBERSORT analysis. **(D)** Pathway analysis of genes down-regulated in cluster 2 *vs.* cluster 1, based on unsupervised clustering of patients with early-stage CRC. **(E)** Pathway analysis of genes up-regulated in cluster 2 *vs.* cluster 1, based on unsupervised clustering of patients with early-stage CRC. Groups with high and low cell numbers were compared with log-rank test.

The unsupervised clustering analysis, using CIBERSORT, yielded two distinct clusters for all patients and patients with early-, late-stage, and metastatic CRC (**Supplementary Figure 1**; **Figure 6A**). While these clusters differed in their immune cell landscape (**Supplementary Figure 1**; **Figure 6B**), we did not observe a significant difference in overall survival when clustering all patients (HR = 0.6762, 95% CI [0.3961-1.1543], p = 0.1621) (**Supplementary Figure 1C**) and patients with late-stage CRC (HR = 0.9975, 95% CI [0.4935-2.0160], p = 0.9944) (**Supplementary Figure 1I**). However, as shown in **Figure 6C**, we did observe a significant difference in overall 5-year survival in patients in cluster 1 vs. cluster 2, in early-stage CRC (HR = 3.0906, 95% CI [1.1940-7.9996], p = 0.03745). As shown in **Figure 6D**, these two clusters significantly differed in abundance of macrophages (M0 and M1), resting and activated mast cells, resting dendritic cells, plasma cells, follicular helper T cells, CD4+ activated memory T cells, CD8+ T cells, naïve B cells, monocytes, neutrophils, activated and resting NK cells and gamma delta T cell types. Specifically, patients in cluster 1 had significantly larger fractions of M0 macrophages, activated mast cells, neutrophils and resting NK cells. These patients also had significantly smaller fractions of M1 macrophages, resting mast cells, resting dendritic cells, plasma cells, follicular helper T cells, CD4+ memory T cells, CD8+ T cells, naïve B cells, monocytes, activated NK cells and gamma delta T cells.

Furthermore, using the immune cell compositions generated by MCP-counter, we also performed unsupervised clustering analysis on all patients and patient with early-, late-stage and metastatic CRC, independently. All of the clusters differed in their immune cell landscape (**Supplementary Figure 2**), however we did not observe a significant difference in overall survival when clustering all patients (HR = 0.9017, 95% CI [0.5265-1.6791], p = 0.7105) and patients with late-stage CRC (HR = 0.9887, 95% CI [0.5043-1.9384], p = 0.9737) (**Supplementary Figure 2C**, I). We observed non-significant difference in overall 5-year survival in patients in cluster 1 vs. cluster 2, in early-stage CRC (HR = 0.3475, 95% CI [0.1341-0.9008], p = 0.0528) (**Supplementary Figure 2F**). As shown in **Supplementary Figure 2E**, these two clusters significantly differed in abundance of B cells, CD8+ T cells, cytotoxic lymphocytes, endothelial cells, fibroblasts, monocytes, myeloid dendritic cells, neutrophils, NK cells and T cell types with patients in cluster 1 showing a higher level of infiltration of each cell type when compared to those in cluster 2.

Furthermore, using immune cell compositions based on the xCell analysis, we performed unsupervised clustering analysis on all patients, and patients with early- and late-stage CRC, independently. Each cluster differed in immune cell composition (**Supplementary Figures 3B, E, H**), however no significant associations were observed in survival of all individuals (HR = 1.0564, 95% CI [0.5710-1.9544], p = 0.8631), early-stage CRC (HR = 0, 95% CI (0–0), p = 0.4601) and late-stage CRC (HR = 0.7174, 95% CI [0.3065-1.6791], p = 0.4905), as shown in **Supplementary Figures 3C, F, I**. This is likely because the unsupervised cluster analysis was performed on all 64 cell types.

### Differential gene expression analysis of unsupervised clusters

The CIBERSORT unsupervised cluster analysis for patients with early-stage CRC resulted in clusters with significantly different survival. Therefore, we sought to determine what genes were differentially expressed between the clusters. Pathway analysis, based on the differentially expressed genes revealed distinct up-regulation and down-regulation of pathways in each cluster. Specifically, shown in **Figure 6D**, pathways involved in extracellular matrix organization, and cell adhesion were significantly up-regulated in cluster 2, compared to cluster 1. Additionally, pathways involved in immune response, including B cell activation, were significantly down-regulated in cluster 2, compared to cluster 1 (**Figure 6E**).

## Discussion

There is growing evidence of a dynamic interaction between cancer cells and immune cells within the tumour microenvironment, and how this plays a crucial role in disease progression (35). The composition of immune cells within the tumour has a profound impact on tumour behaviour and can affect therapeutic responses (36). We therefore sought to elucidate the distribution of leukocytes within the colorectal tumour microenvironment, as this may represent an active engagement of the immune system in this cancer.

This study used three transcriptome-based computational approaches, CIBERSORT, xCell, and MCP-counter, to quantify the abundance of immune cells within the CRC tumour microenvironment. CIBERSORT utilizes a deconvolution algorithm that relies on a reference matrix to estimate fractions of 22 immune cell types (21). xCell is a method that utilizes gene signatures and deconvolution to quantify the abundance of 64 immune cell types (24). MCP-counter utilizes transcriptomic markers expressed in a cell population to quantify the abundance of 10 cell types within a sample (22). These methods use arbitrary units to score cell type abundance within a bulk tumour (37). Our study demonstrates similarities in analysis of immune cell composition when comparing each method. Comparing immune cell abundance in CRC tissue *vs.* normal adjacent tissue, we found similar trends with respect to abundance of B cell lineage across computational methodologies. This is consistent with Sturm et al., who previously reported a high correlation of B cell estimates using these methods (37). Our results also show high correlation in dendritic cell abundance predicted by CIBERSORT and xCell, but not MCP-counter.

All three methodologies indicate that the immune cell landscape within CRC tissue differs significantly from normal adjacent colon tissue. The majority of immune cell types are present in lower abundance in the tumour microenvironment compared to normal colon tissue, reinforcing the existence of immune “cold” tumours. Our cluster analysis revealed stark differences in immune cell composition, which is especially observed in the unsupervised cluster analysis based on MCP-counter, clearly outlining a population of “cold” tumours. However, these drastic distinctions do not translate to significant differences in overall survival. The unsupervised clustering based on CIBERSORT does, however, suggest that a more comprehensive analysis of the immune cell composition may reveal a distinct population with a survival advantage. Specifically, our results indicate that a subpopulation of individuals with early-stage CRC have significantly better prognosis, if their tumours have a higher abundance of immune cell infiltration. Although the overall abundance of immune cell composition does not correlate to survival benefit with late-stage CRC or metastatic CRC, we did find specific immune cell types (dendritic cells) that provided a survival advantage.

Tumour infiltrating leukocytes have been characterized as both pro-tumourigenic and anti-tumourigenic (38). In general, studies have demonstrated that immune infiltration is correlated with better prognosis in cancers, including CRC (12, 39). Importantly, our results indicate an inverse correlation between overall survival and absolute leukocyte abundance, as measured by CIBERSORT (**Figure 2**). It is noteworthy that the Immunoscore derived from the xCell analysis was not correlated to overall survival. While the total immune landscape should be considered, it is apparent that infiltration of specific cell types has a more profound influence on overall survival and prognosis. For example, total infiltration of CD3^+^ and CD8^+^ T cells have been used as measure of prognosis in CRC (39). This method is determined by scoring densities of CD3^+^ and CD8^+^ staining in colorectal tumours and their invasive margins. Furthermore, Diederichsen et al. demonstrated that a low ratio of CD4^+^/CD8^+^ cells was associated with better prognosis in patients with CRC (34). Collectively, these studies used immunohistochemical staining to identify lymphocyte infiltration. Consistent with these studies, CD8^+^ T cells were correlated with better overall survival in metastatic CRC, based on xCell. However, inconsistent with previous studies, our study identified CD8^+^ T cells as negative predictors of prognosis in patients with early-stage CRC, based on both CIBERSORT and xCell analysis. Additionally, we demonstrated that the prognostic impacts of plasma cells and mast cells were also dependent on tumour stage. The dependence on tumour stage may indicate that disease progression, resulting in metastatic lesions, yields contributions of the metastatic tumour microenvironment that are not accounted for in the studies of the primary tumour. Interestingly, the majority of studies have demonstrated that mast cells promote tumour angiogenesis in various cancers by secreting pro-angiogenic factors such as VEGF, bFGF and IL-8 (40). Additional studies have suggested that mast cells can act as both pro- and anti-tumourigenic (41). Importantly, in our study mast cells emerged as a positive prognostic marker in late-stage CRC, (predicted by both CIBERSORT and xCell). This is in keeping with a study in prostate cancer which demonstrates that the role of mast cells is dynamic and dependent on tumour stage (42). Further research is warranted to address the stage-dependent role of mast cells in CRC.

In our study, dendritic cells were found to consistently have a significant impact on prognosis in all patients and across methodologies. Dendritic cells are antigen-presenting cells and are functionally important for the induction of a coordinated immune response, which results in the activation and expansion of cytotoxic T cells (43). While activated dendritic cells are shown to have a positive impact on prognosis, immature dendritic cells were also found to be associated with better overall survival, specifically in metastatic CRC. This is intriguing as immature dendritic cells are shown as immunosuppressive and accumulation of these cells suggests inhibition of dendritic cell maturation by chemokines present in the tumour microenvironment, including vascular endothelial growth factor and interleukins such as IL-6 and IL-10 (44, 45). Studies of other cancers have shown that tumour-associated dendritic cells are impaired in antigen up-take and presentation, demonstrating that the role of immature dendritic cells differs from their activated counterparts.

Importantly, chemotherapies and immunotherapies have been shown to influence and change the immune cell landscape in colorectal cancer. Specifically, 5-fluorouracil depletes myeloid-derived suppressor cells, resulting in an antitumor response (46). Intriguingly, Cetuximab has been shown to increase CD8^+^ T cells and promote cytotoxic activity in colorectal cancer (47). Additionally, oxaliplatin treatment significantly increased CD8^+^ T cell infiltration in a murine colon cancer model (48). These findings suggest that stage-related differences observed in immune cell composition, may be influenced by specific therapies used to treat each patient. Therefore, the impact of chemotherapy and immunotherapy treatments on immune cell infiltration in colorectal cancer should be further explored.

Collectively, using gene expression analysis, we have evaluated the prognostic value of the immune cell landscape in CRC, utilizing three computational methods. We have demonstrated that differences in the immune cell infiltration of CRC infer prognostic value. We also demonstrated that influences on prognosis by specific cell types are dependent on tumour stage. Further research is warranted to assess whether these cell types can be used to implement an immunoscore that can be used in clinical practice. Further research is also warranted to understand whether the presence or absence of these cell types can predict response to immunotherapies and other treatments at different stages of cancer progression. We anticipate that this research offers potential targets for immunotherapy, to aid the therapeutic process and improve overall survival of CRC patients.

## Data availability statement

Publicly available datasets were analyzed in this study. POG data can be found here: https://ega-archive.org, accession; EGAS00001001159 and TCGA can be found here: https://www.cancer.gov/tcga.

## Ethics statement

The studies involving human participants were reviewed and approved by UBC Human Ethics. The patients/participants provided their written informed consent to participate in this study.

## Author contributions

ZB, PZ, and IT designed the study. SS and PZ collected and analyzed the data. ZB and SS interpreted the data. ZB, SS, PZ and IT wrote the manuscript. All authors contributed to the article and approved the submitted version.
