# Supplementary material for: Characterization of the immune cell landscape in CRC: Clinical implications of tumour-infiltrating leukocytes in early- and late-stage CRC
Source: Front Immunol. 2023 Feb 8;13:978862. doi: 10.3389/fimmu.2022.978862 (PMC9945970; doi:10.3389/fimmu.2022.978862)
Supplement: Supplementary file 1 [file DataSheet_1.docx]

**Characterization of the immune cell landscape in CRC: clinical implications of tumour-infiltrating leukocytes in early- and late-stage CRC**

Zainab A. Bazzi^1,2*^, Sophie Sneddon^2*^, Peter G. Y. Zhang^1,2^, George C. Chang^1,2^, Isabella T. Tai^1,2^

**AUTHOR AFFILIATIONS:**

^1^Division of Gastroenterology, Department of Medicine, University of British Columbia, Vancouver, British Columbia, Canada

^2^Canada’s Michael Smith Genome Sciences Centre, BC Cancer, Vancouver, British Columbia, Canada

*ZAB and SS contributed equally to this work.

**Supplementary Material**

| **Supplementary Table 1.** *Clinicopathological characteristics of patients from TCGA and POG cohorts evaluated by CIBERSORT.* | | |
| --- | --- | --- |
| **Patient Characteristics** | **TCGA** | **POG** |
|  |  |  |
|  | Number of Patients (percent) | Number of Patients (percent) |
| **Total** | 308 (100) | 54 (100) |
| **Age** |  |  |
| <45 | 21 (6.8) | 7 (13.0) |
| 45-59 | 70 (22.7) | 21 (38.9) |
| 60-80 | 172 (55.8) | 25 (46.3) |
| >80 | 43(14.0) | 0 |
| Not Reported | 2 (0.7) | 1 (1.9) |
| **Sex** |  |  |
| Male | 165 (53.6) | 27 (50.0) |
| Female | 142 (46.1) | 27 (50.0) |
| Not Reported | 1 (0.3) | 0 |
| **Race** |  |  |
| White | 161 (52.3) | N/A |
| Black or African American | 33 (10.7) | N/A |
| Asian | 6 (1.9) | N/A |
| Not Reported | 108 (35.1) | N/A |
| **Body Mass Index (BMI)** |  |  |
| < 24.9 | 56 (18.2) | N/A |
| 24.9-30 | 67 (21.8) | N/A |
| > 30 | 40 (13.0) | N/A |
| Not Reported | 145 (47.0) | N/A |
| **Tumour Stage** |  |  |
| I | 52 (16.8) | 0 |
| II | 117 (38.2) | 0 |
| III | 89(28.9) | 0 |
| IV | 38 (12.5) | 54 (100) |
| Not Reported | 12 (3.6) | 0 |
| **Tumour Site** |  |  |
| Right-sided | 83 (26.9) | N/A |
| Left-sided | 124 (40.3) | N/A |
| Rectal | 36 (11.7) | N/A |
| Not Specified | 65 (21.1) | N/A |

**Supplementary Figure 1.** *Unsupervised clustering of patients with CRC, based on immune cell infiltration, using CIBERSORT analysis.* A) Unsupervised clustering of all patients with CRC, based on immune cell infiltration, using CIBERSORT analysis. B) Heatmap of immune cell infiltration, of patients in cluster 1 and 2, based on CIBERSORT analysis. C) Kaplan-Meier curve for overall five-year survival for clusters 1 and 2, resulting from unsupervised clustering for all CRC patients, based on CIBERSORT analysis. D) Unsupervised clustering of late-stage CRC patients, based on immune cell infiltration, using CIBERSORT analysis. E) Heatmap of immune cell infiltration, of patients in cluster 1 and 2, based on CIBERSORT analysis. F) Kaplan-Meier curve for overall five-year survival for clusters 1 and 2, resulting from unsupervised clustering for late-stage CRC patients, based on CIBERSORT analysis. G) Unsupervised clustering of metastatic CRC patients, based on immune cell infiltration, using CIBERSORT analysis. H) Heatmap of immune cell infiltration, of patients in cluster 1 and 2, based on CIBERSORT analysis. I) Kaplan-Meier curve for overall five-year survival for clusters 1 and 2, resulting from unsupervised clustering for metastatic CRC patients, based on CIBERSORT analysis. Groups with high and low cell numbers were compared with log-rank test.

**Supplementary Figure 2.** *Unsupervised clustering of patients with CRC, based on immune cell infiltration, using MCP-counter analysis.* A) Unsupervised clustering of all patients with CRC, based on immune cell infiltration, using MCP-counter analysis. B) Heatmap of immune cell infiltration, of patients in cluster 1 and 2, based on MCP-counter analysis. C) Kaplan-Meier curve for overall five-year survival for clusters 1 and 2, resulting from unsupervised clustering for all CRC patients, based on MCP-counter analysis. D) Unsupervised clustering of early-stage CRC patients, based on immune cell infiltration, using MCP-counter analysis. E) Heatmap of immune cell infiltration, of patients in cluster 1 and 2, based on MCP-counter analysis. F) Kaplan-Meier curve for overall five-year survival for clusters 1 and 2, resulting from unsupervised clustering for early-stage CRC patients, based on MCP-counter analysis. G) Unsupervised clustering of late-stage CRC patients, based on immune cell infiltration, using MCP-counter analysis. H) Heatmap of immune cell infiltration, of patients in cluster 1 and 2, based on MCP-counter analysis. I) Kaplan-Meier curve for overall five-year survival for clusters 1 and 2, resulting from unsupervised clustering for late-stage CRC patients, based on MCP-counter analysis. J) Unsupervised clustering of metastatic CRC patients, based on immune cell infiltration, using MCP-counter analysis. K) Heatmap of immune cell infiltration, of patients in cluster 1 and 2, based on MCP-counter analysis. L) Kaplan-Meier curve for overall five-year survival for clusters 1 and 2, resulting from unsupervised clustering for metastatic CRC patients, based on MCP-counter analysis. Groups with high and low cell numbers were compared with log-rank test.

**Supplementary Figure 3.** *Unsupervised clustering of patients with CRC, based on immune cell infiltration, using xCell analysis.* A) Unsupervised clustering of all patients with CRC, based on immune cell infiltration, using xCell analysis. B) Heatmap of immune cell infiltration, of patients in cluster 1 and 2, based on xCell analysis. C) Kaplan-Meier curve for overall five-year survival for clusters 1 and 2, resulting from unsupervised clustering for all CRC patients, based on xCell analysis. D) Unsupervised clustering of early-stage CRC patients, based on immune cell infiltration, using xCell analysis. E) Heatmap of immune cell infiltration, of patients in cluster 1 and 2, based on xCell analysis. F) Kaplan-Meier curve for overall five-year survival for clusters 1 and 2, resulting from unsupervised clustering for early-stage CRC patients, based on xCell analysis. G) Unsupervised clustering of late-stage CRC patients, based on immune cell infiltration, using xCell analysis. H) Heatmap of immune cell infiltration, of patients in cluster 1 and 2, based on xCell analysis. I) Kaplan-Meier curve for overall five-year survival for clusters 1 and 2, resulting from unsupervised clustering for late-stage CRC patients, based on xCell analysis. J) Unsupervised clustering of metastatic CRC patients, based on immune cell infiltration, using xCell analysis. K) Heatmap of immune cell infiltration, of patients in cluster 1 and 2, based on xCell analysis. L) Kaplan-Meier curve for overall five-year survival for clusters 1 and 2, resulting from unsupervised clustering for metastatic CRC patients, based on xCell analysis. Groups with high and low cell numbers were compared with log-rank test.
