# Supplementary figures and images for: Characterization of the immune cell landscape in CRC: Clinical implications of tumour-infiltrating leukocytes in early- and late-stage CRC
Source: Front Immunol. 2023 Feb 8;13:978862. doi: 10.3389/fimmu.2022.978862 (PMC9945970; doi:10.3389/fimmu.2022.978862)

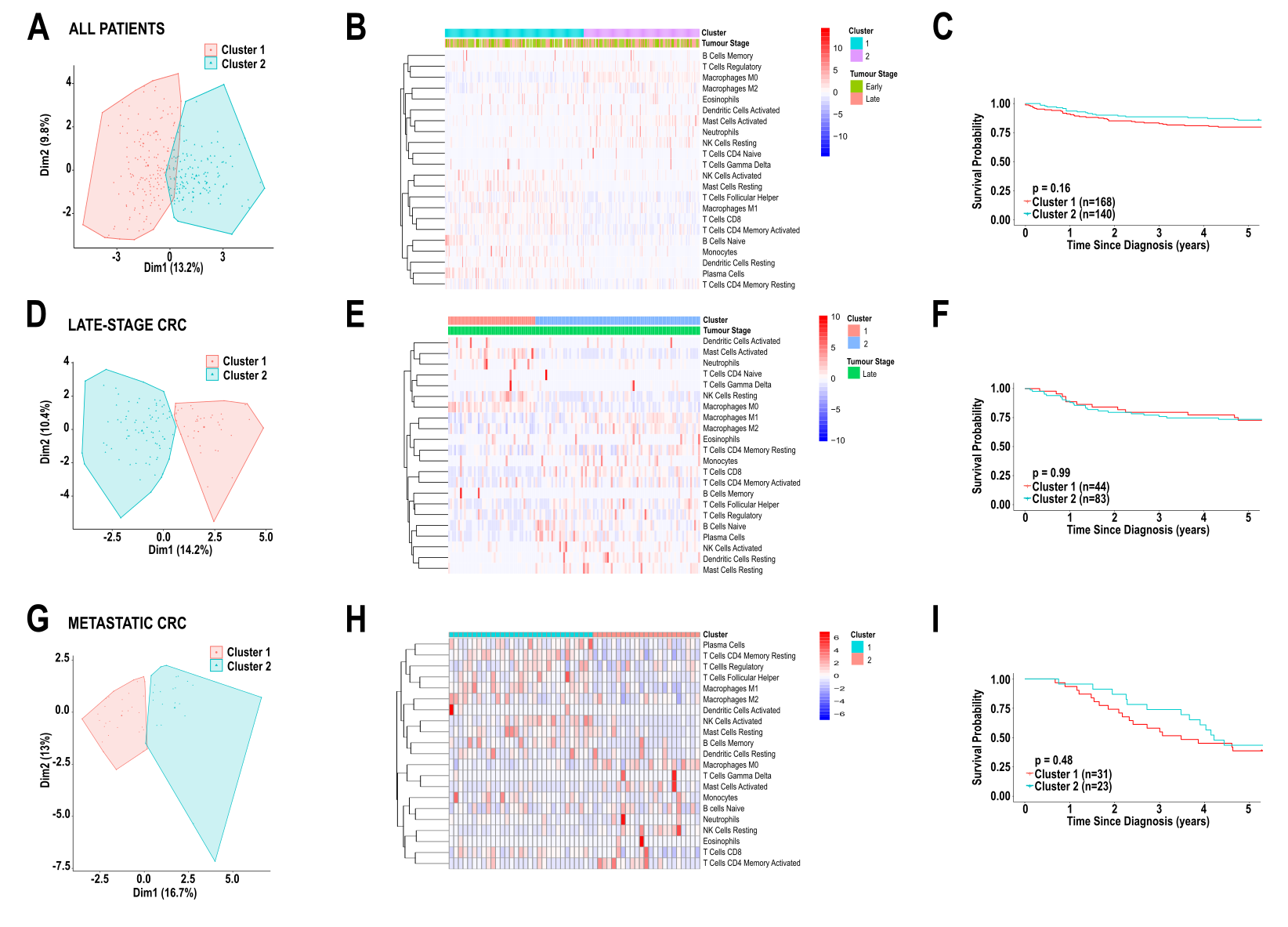

Supplement: Supplementary file 2 [file Image_1.png]

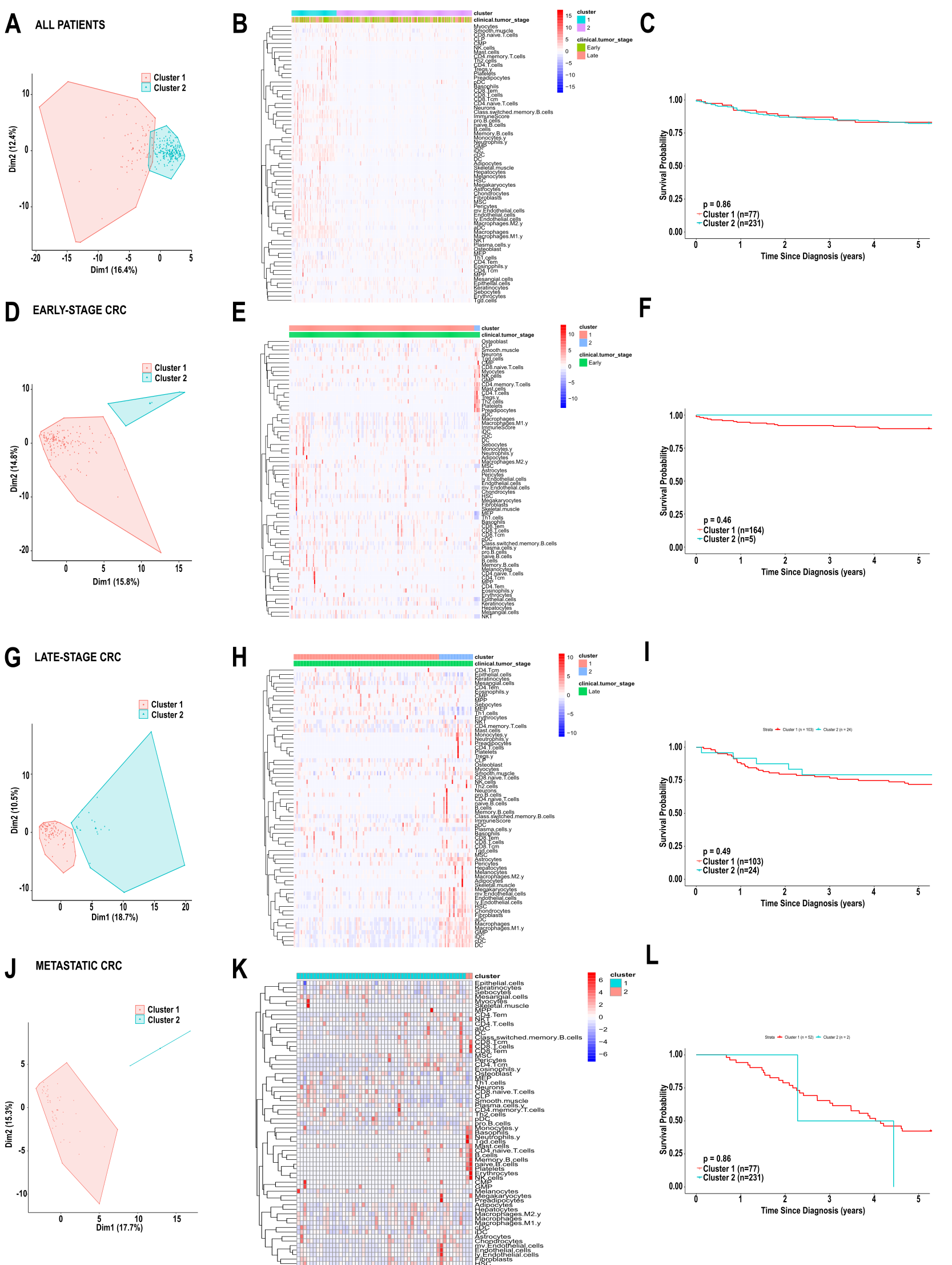

Supplement: Supplementary file 4 [file Image_3.png]
